# Optimization of Culture Conditions and Production of Bio-Fungicides from *Trichoderma* Species under Solid-State Fermentation Using Mathematical Modeling

**DOI:** 10.3390/microorganisms9081675

**Published:** 2021-08-06

**Authors:** Afrasa Mulatu, Tesfaye Alemu, Negussie Megersa, Ramesh R. Vetukuri

**Affiliations:** 1 Department of Microbial, Cellular and Molecular Biology, Addis Ababa University, Addis Ababa P.O. Box 1176, Ethiopia; afrasa.mulatu@aau.edu.et (A.M.); tesfaye.alemu@aau.edu.et (T.A.); 2 Department of Chemistry, Addis Ababa University, Addis Ababa P.O. Box 1176, Ethiopia; negussie.megersa@aau.edu.et; 3 Department of Plant Breeding, Swedish University of Agricultural Sciences, 230 53 Alnarp, Sweden

**Keywords:** agro-industrial waste, bioformulation, response surface methodology, viability, wheat bran

## Abstract

Agro-industrial wastes suitable for economical and high mass production of novel *Trichoderma* species under solid-state fermentation were identified by optimizing the culture conditions using a mathematical model and evaluating the viability of the formulated bio-product. Fourteen inexpensive, locally available, organic substrates and cereals were examined using a one-factor-at-a-time experiment. The fungus colonized nearly all substrates after 21 days of incubation, although the degree of colonization and conidiation varied among the substrates. A mixture of wheat bran and white rice (2:1 *w/w*) was found to support maximum growth of *T. asperellum* AU131 (3.2 × 10^7^ spores/g dry substrate) and *T. longibrachiatum* AU158 (3.5 × 10^7^ spores/g dry substrate). Using a fractional factorial design, the most significant growth factors influencing biomass production were found to be temperature, moisture content, inoculum concentration, and incubation period (*p ≤* 0.05). Analysis of variance of a Box–Behnken design showed that the regression model was highly significant (*p ≤* 0.05) with *F*-values of 10.38 (*P* = 0.0027, *T. asperellum* AU131) and 12.01 (*p* < 0.0017, *T. longibrachiatum* AU158). Under optimal conditions, maximum conidia yield of log_10_ (8.6) (*T. asperellum* AU131) and log_10_(9.18) (*T. longibrachiatum*) were obtained. For wettable powder *Trichoderma* species formulations, it was possible to maintain conidial viability at room temperature (25 °C) for eight months at concentrations above 10^6^ CFU/g.

## 1. Introduction

*Trichoderma* is a versatile genus of fungi that has agricultural as well as industrial importance. It is one of the most widespread biological agents currently used in agriculture to control different plant diseases [1,2]. It is present in more than 60% of registered biological pesticides worldwide. No other beneficial fungus in the agriculture field has received as much combined attention from science and the commercial market [2]. *Trichoderma* species can promote plant growth and productivity, help to manage pests and pathogens, alleviate abiotic stresses, biodegrade xenobiotic compounds, and produce industrially important metabolites. Using biocontrol agents is one of the sustainable approaches for crop cultivation with numerous benefits, including increased disease protection and yield, as well as reduced chemical impact on the environment [3,4,5,6,7].

The development of new biocontrol agent products to control plant pathogens requires large scale screening of candidate antagonists, developing mass production protocols that optimize product quantity and quality, and devising a product formulation that preserves, aids product delivery and enhances bioactivity [8,9]. The establishment of robust technology is key for manufacturing microbial biopesticides. Formulation-based solutions related to challenges in terms of biocontrol agent stability, efficacy, and application have been addressed by evaluating the impact of formulation ingredients and processes on the physical characteristics, biological activity, storage stability and field efficacy of selected biocontrol agents [10,11,12]. It is important in the manufacturing process to ensure protection of the active ingredients (conidia or mycelia of antagonist fungi) against conditions of extreme pH, low humidity, chemicals, and UV radiation. The development of a reliable biocontrol agent requires identification of a proper formulation to overcome environmental limitations and give the antagonist a competitive advantage over pathogens and other microflora. The formulations can be designed to include nutrients important for the biocontrol agent’s growth, osmoregulation and initial growth from dried biomass [13,14]. Moreover, the biocontrol agents must survive several processing steps, including harvesting, drying, formulation, storage, and delivery.

Developmental costs and technological challenges are major hindrances to the development of successful products [15,16]. Cost-effective large-scale production can be achieved through solid-state fermentation (SSF). Thus, increasing demands for bio-fungicide production to replace excessively used chemical pesticides have recently enhanced interest in SSF technology. SSF simulates the natural habitat of fungi and is, therefore, the preferred choice for these microorganisms to grow and produce useful value-added products [17]. It is a cost-effective process widely used for the mass production of filamentous fungi, their enzymes and/or other metabolites on solid substrates with sufficient moisture but not in the free state [18]. The raw materials used as an organic substrate for biomass production account for 35–40% of production costs [19]. Therefore, the utilization of agro-industrial wastes that are cheap, easily available and support extensive growth of *Trichoderma* is required for the production of value-added products. It provides avenues for the safe utilization of wastes while reducing the cost and environmental pollution load of waste disposal. In recent years, the global production, registration, and application of biological pesticides in agriculture as alternatives to chemicals have rapidly increased owing to public concerns about human health, food safety and the impact on the environment [20,21]. Recent literature surveys have shown that the number of *Trichoderma*-containing products on the international market has grown exponentially, with more than 300 products now available [22,23]. Currently, there are eight registered products based on the genus *Trichoderma* in Brazil [24] and more than 250 commercial formulations in India [25], whereas there is no registered product in Ethiopia. This highlights the lack of formulated products commercially available in Ethiopia. One developed product has been tested for the control of coffee wilt disease (CWD) caused by *Fusarium xylarioides* and shown to be effective and efficient under greenhouse and field conditions in Ethiopia (unpublished data). Introducing biological control agents as part of a *F. xylarioides* control strategy is highly desirable, especially because there is a lack of an efficient synthetic fungicide. 

Different *Trichoderma* species require specific culture conditions for maximum conidia productivity, and hence no defined medium is available for optimum conidia production. Mathematical modeling is a useful approach for optimizing culture conditions with fewer experiments than conventional methods. Experimental design can be regarded as a process by which certain factors are selected and deliberately varied in a controlled manner to obtain their effects on a response of interest, often followed by the analysis of the experimental results. Several modeling and optimization methodologies are available ranging from simple models like one factor at a time (OFAT) to complex statistical designs such as two-level fractional factorial design (FFD), Box–Behnken design (BBD), and response surface methodology (RSM) [26,27]. OFAT is a traditional method employed for screening substrates and growth factors. This method has several disadvantages, such as time consumption, huge resource requirements, less capable of finding true optimum levels due to the interactions among factors and a several-fold increase in the number of experiments. A better alternative to OFAT is FFD, which can be employed for screening significant factors at different levels, with advantages of a better yield, reproducible results and better design space for experimental trials [26]. Moreover, statistically designed experiments could effectively solve such issues and minimize the error in determining the effect of factors and interaction between factors [27,28]. The design of the experiment (DOE) offers a reduced number of experiments and increased process efficiency [29].

Response surface methodology (RSM) is a collection of mathematical and statistical tools for designing experiments, developing models, evaluating the effects of factors and identifying optimum conditions of factors for desirable responses [27]. It can be used to evaluate and predict interactions among different process parameters. The Box–Behnken design (BBD) is a second-order multivariate technique based on three-level partial factorial designs [29]. It enables the estimation of parameters in a quadratic model and evaluation of the lack of fit of a model. This methodology was applied in the present study to identify the optimal growth conditions for maximizing conidia production from agro-industrial wastes. The optimization process involved three major steps: statistically designed experiments, estimation of the coefficients in a mathematical model, and prediction of the response to check the adequacy of the model [28,30]. Physical parameters such as the initial moisture content, cultivation time, and temperature greatly influence the SSF process [31]. In this study, these three parameters were considered for the optimization of *Trichoderma* species conidia production and to find interaction effects among these variables. 

Therefore, the present study was undertaken to find suitable agro-industrial wastes for economical and high mass production of novel *Trichoderma* species under SSF by optimizing the culture conditions using a mathematical model and determining the viability of the formulated bio-product under different storage conditions.

## 2. Materials and Methods

### 2.1. Sources of Trichoderma Species

*Trichoderma* species were isolated from *Coffea arabica* rhizospheric soils from Ethiopia. The species were identified based on morphological characteristics and sequences of two genes (*tef1-*α gene and ITS region of rRNA) [32,33], but due to possible commercial interests, they have not yet been deposited in a public database (unpublished data). The species were grown on potato dextrose agar (PDA; Hi-Media, Mumbai, India) at 25 ± 2 °C in 90 mm Petri plates. For the inoculum suspension, spores were harvested from the surface by pouring sterile 0.1% Tween-80 to wash off the conidia [34]. The conidia concentration was measured and adjusted by counting with a hemocytometer (Neubauer chamber, Taufkirchen, Germany) under an Olympus BX41 phase-contrast microscope (Sigma-Aldrich Chemie GmbH, Taufkirchen, Germany) (400×). Each solid substrate was inoculated with an inoculum level (spore suspension) of 1 × 10^7^ spores/mL. 

### 2.2. Sreening of Organic Substrates Using the OFAT Method

**Figure 1:**
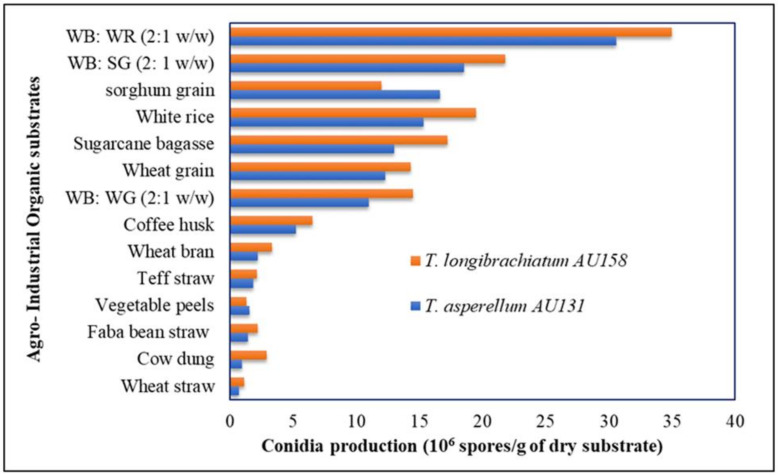
Spore production of *Trichoderma* species on different agro-industrial wastes and cereals after 21 days of incubation at 25 ± 2 °C (WB = wheat bran, WR = white rice, SG = sorghum grain and WG = wheat grain).

**Figure 2:**
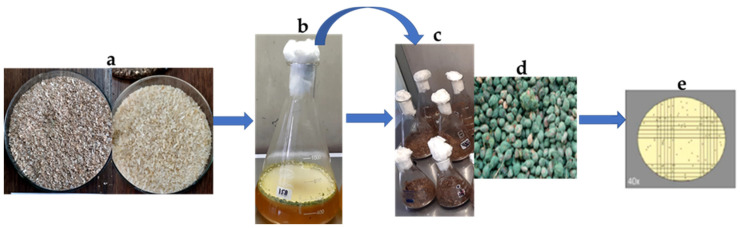
Schematic representation of the SSF system for 21 days, (**a**) Organic substrates (wheat bran: rice (2:1 *w*/*w*) preparation and sterilization at 121 °C, (**b**) Inoculum preparation, (**c**) Inoculation, (**d**) Sporulation (conidia): (**e**) Counting of spores/g dry substrate using haemocytometer.

Eleven locally available and low-cost organic substrates considered as agricultural wastes, by-products and three cereal grains mixtures with wheat bran were included in the study (Figure 1): wheat straw, faba bean straw, vegetable peels, teff straw, and cow dung (agro-wastes); wheat bran, coffee husks, and sugarcane bagasse (industrial byproducts); and mixture of white rice and wheat bran, wheat and wheat bran, and sorghum grains and wheat bran (cereals). The organic substrates were examined separately or in combination by adding each substrate into flasks for OFAT experiments. For biomass production of *Trichoderma* species, all substrates were soaked in tap water overnight and excess water was drained out [13]. SSF was carried out in 500 mL flasks containing 50 g (dry weight) of a solid substrate supplemented with 1% (*v*/*v*) glycerol and 1% (*w*/*v*) (NH_4_)_2_SO_4_ as a nitrogen source. The flasks were sterilized at 121 °C for 15 min and, after cooling, inoculated with 5 mL of spore suspension (1 × 10^7^ spores/mL), followed by incubation at 25 ± 2 °C for 21 days [13]. The initial moisture content was adjusted to 65% [35]. All liquid added to the flasks was taken into consideration in calculating the moisture content. The experiment was performed in triplicate. After incubation, the *Trichoderma* species inoculum was removed from the flasks, dried in a fluid bed drier and the spores were counted (Figure 2). The best spore producing substrates were selected for further experiments.

### 2.3. Statistical Optimization of Conidia Production under SSF

#### 2.3.1. Identification of Significant Factors Using a Two-Level FFD

Based on the preliminary results from OFAT experiments, combination of two substrates was identified to have strong effects on conidia production. A mixture of wheat bran and white rice (1:1 *w*/*w*) was selected for screening and optimization of critical growth factors. Factors such as moisture content, temperature, inoculum concentration, incubation period, inoculum age and pH were assessed. Each variable was represented at two levels, high and low denoted by (+1) and (−1), respectively, and a center point (0) (Table 1). A FFD matrix consisting of a total of 20 experimental trials (16 trials for design and 4 replicates at the center points) generated by the software was applied for screening and optimization of critical growth factors (Table 2). A first-order polynomial model was used to screen many independent variables and suggest fewer variables for further optimization of the two-level factorial design, allowing investigation of *n*−*1* variables with at least *n* experiments. The main effect was calculated as the difference between the average of measurements made at a high setting (+1) and the average of measurements observed at a low setting (−1) for each factor. The variables that significantly affected conidia production were considered for further optimization using the statistical model of Box–Behnken RSM.

#### 2.3.2. Optimization of Significant Variables Using BBD 

For both *Trichoderma* species, three critical parameters were identified to have strong effects on the response (conidia production) using two-level FFD experiments. The moisture content, incubation temperature and incubation period significantly affected conidia production of *T. asperellum* AU131, whereas moisture content, incubation temperature, and inoculum concentration significantly affected conidia production of *T. longibrachiatum* AU158. To determine the individual and interactive effects of these medium components for conidia production, a total of 17 experimental fermentation groups were used [36]. The experimental design comprised 17 tests at low (−1), medium (0) and high (+1) levels of all three factors. The independent variables (*A*, *B*, and *C*) and their levels, actual values, and coded values are presented in Table 3 and Table 4. 

For each test, 50 g of wheat bran and white rice (2:1 *w*/*w*) was initially sterilized at 121 °C for 15 min. Then, after cooling to room temperature, each flask was filled with a sterile substrate in a laminar flow cabinet under sterile conditions. The final moisture content was adjusted to 50%, 65%, or 80% (*w*/*w*) by inoculating the solid substrate with a spore suspension (1 × 10^7^ spores/g dry matter) in distilled water containing 1% (*v*/*v*) glycerol and 1% (*w*/*v*) (NH_4_)_2_SO_4_ [28,34]. The pH value of the wet substrate was approximately 6.7. The moisture content of the solid substrate was adjusted by drying 50 g of wheat bran and rice grains (3:1 *w*/*w*) to a constant weight at 90 °C. The spore count was determined at regular intervals of time as described below and then logarithmically transformed before statistical analysis.

A BBD matrix consisting of a total of 17 experimental trials (12 trials for design and 5 replicates at the center points) generated by the software was applied to evaluate the response pattern and determine the optimum combination of variables for both *Trichoderma* species (Table 5 and Table 6).

For predicting the optimal point, a second-order polynomial model was fitted to correlate the relationship between the independent variables and response. The following second-order polynomial equation describes the relationship between the dependent and independent variables:

*Y* = β_0_ + β_1_A + β_2_B +β_3_C +β_11_A^2^ + β_22_B^2^ +β_33_ C^2^ + β_12_ AB + β_13_ AC + β_23_ BC

where *Y* is the predicted response (biomass production (log_10_ (*Y*) conidia/g dry substrate), β_0_ is the intercept, β_1_, β_2_, and β_3_ are linear coefficients, β_1__1_, β_22_, and β_33_ are the squared coefficients, and β_12_, β_13_, and β_23_ are the interaction/quadratic coefficients. The polynomial model was evaluated using various statistical analysis parameters, i.e., *p*-value, *F*-test, adjusted determination of coefficient (*R*^2^ adj), which measures the signal-to-noise ratio, and coefficient of determination (*R*^2^), to assess the goodness-of-fit of the developed quadratic mathematical model to the experimental data. The quality of fit of the polynomial model equation was expressed by the coefficient of determination (*R*^2^).

### 2.4. Determination of Spore Production on Different Organic Substrates

Conidia production of *Trichoderma* species on all substrates was examined by the serial dilution method. Three independent 1 g samples of the colonized substrates were used. Each sample was added to 9 mL of sterile deionized water and the surfactant Tween 80 (0.05% *v*/*v*). Before shaking, glass beads were used to remove the conidia attached to the surface of the substrate [13]. Suspensions were vigorously shaken with the aid of vortex mixer for 1 min, filtered through two layers of muslin cloth, and then serial dilutions were made [31]. Conidia were counted under an Olympus BX41 phase-contrast microscope (JPK Instruments, Berlin, Germany) at high resolution (400×) in the middle square of a hemocytometer (Neubauer chamber, Germany) as conidia/g dry substrate. Finally, the spore production values (*Y*) were logarithmically transformed before statistical analysis (log_10_(*Y*)).

### 2.5. Formulation of Bio-Fungicides

Wettable powder-based formulations of *Trichoderma* species were prepared using talc powder as a carrier substrate based on its use in previous studies [37,38,39,40,41]. Talc-based formulations of the biocontrol agents were studied under the optimum culture conditions predicted by BBD in the SSF system. Wheat bran and white rice (2:1 *w*/*w*) were used as an organic substrate and selected based on the FFD results. Then, one hundred gram of each carrier material was placed in a metal tray under aseptic conditions and the pH was adjusted to 7 by adding CaCO_3_ at 15 g/kg [42]. Next, 50 g of each colonized substrate of fungal bioagent solubilized with sterile distilled water was added to Erlenmeyer flasks (500 mL) containing 100 g of each carrier. Carboxymethylcellulose (CMC) (10g/kg), Tween 20 (1%), and glycerol (3%) were then added and mixed well [43]. The incubation period for *T. asperellum* AU131, inoculum concentration of *T. longibrachiatum* AU158, and moisture content and temperature for both biocontrol agents were as described by BBD in this study (Section 2.3.1). The flasks were incubated at 25 °C for the optimal period according to BBD until fungal spores covered the surface of the carriers. The dried bio-formulated products were packed into 50 mL sterile, plastic, screw-capped bottles (Falcon Plastics, Brookings, USA) with three replicates for each temperature, then sealed and stored at room temperature (25 °C) or in the refrigerator (4 °C) to assess the viability and shelf life.

### 2.6. Determination of Shelf Life and Viability of Bio-Fungicides

To estimate the number of colony forming units (CFU) of both *Trichoderma* species in the formulation, 1 g of each formulated bioproduct was serially diluted to 10^−5^, and then 0.1 mL of the diluted solution was placed on fresh PDA plates [44]. Five replicates were conducted, and the plates were incubated at 25 ± 2 °C for 3–5 days. At the end of the incubation period, the number of CFUs was determined. Population counts of *Trichoderma* species in the formulated product were recorded at the start (0 month) and then at two-month intervals for 12 months. The shelf life of the formulations during the storage period was expressed as log_10_CFU g^−1^ [45].

### 2.7. Statistical Data Analysis

The screening of organic substrates using the OFAT method was analyzed by R software. The mathematical modeling was carried out and optimal conditions of the SSF process identified using Reliasoft2020 Weibull^++^ software. Design-Expert 12 software (Stat Ease Inc., Minneapolis, MN, USA) was used to generate the design matrix and analyze the results. The upper limit and lower limit for each independent variable studied were based on our preliminary experiments. The conidia/g and CFU values were log-transformed (base 10) and analyzed by analysis of variance (ANOVA). All means of the treatments were compared using Duncan’s multiple range tests at *p* ≤ 0.05. Multiple regression analysis methods were used to study second-order polynomial equations of independent variables. To check the statistical significance of these equations, an *F*-test was applied to evaluate *R*^2^. The significance of independent variables and their interactions were tested using ANOVA analysis. Standardized effects of the independent variables and their interactions on dependent variables were also investigated by preparing a Pareto chart. Results were assessed with various descriptive statistics, i.e., *p*-value, *F*-test, *R*^2^, *R*^2^ adj, sum of squares (SS), and mean sum of squares (MSS) test to assess the goodness-of-fit of the developed quadratic mathematical model to the experimental data [46].

## 3. Results

### 3.1. Colonization and Sporulation of Trichoderma Species

The results of this study showed that both *Trichoderma* species grew on all 14 examined solid organic substrates and abundantly sporulated on them, but the level of colonization, conidiation and production of biomass was significantly different among the substrates (*p ≤* 0.05). After 21 days, *T. asperellum* AU131 and *T. longibrachiatum* AU158 colonized all substrates but to different extents, from scanty growth to covering the whole substrate (Figure 1). The solid media initially had a white color due to development of the mycelium, then the color gradually changed, and finally, green/light green colored spores were observed when fully sporulated. Among the substrates tested, wheat bran combined with white rice (2:1 *w*/*w*) was found to support maximum growth of *T. asperellum* AU131 (3.2 × 10^7^ spores/g dry substrate) and *T. longibrachiatum* AU158 (3.5 × 10^7^ spores/g dry substrate), which was significantly (*p ≤* 0.05) higher than for the other substrates. 

Among the remaining substrates, wheat bran and sorghum grain, wheat bran and wheat grain, sugarcane bagasse, wheat, and sorghum grains showed moderate spore production as biocontrol species, which was not significantly different (Figure 1). Wheat straw showed the least fungal population with scanty colonization and biomass. Coffee husks, cow dung, vegetable peels, teff straw, and wheat straw were abandoned due to poor colonization and conidiation of the biocontrol fungus. The aforementioned moderate and high biomass producing substrates can be recommended as a suitable fermentation media for the mass multiplication of *Trichoderma* species, and the SSF process reported here could be scaled up and developed for cost-effective commercial production of *Trichoderma* based bioproducts. The highest performing substrate, i.e., wheat bran and white rice (2: 1 *w*/*w*), was selected as a suitable substrate for the further identification of significant growth factors and optimization of culture conditions using mathematical modeling.

### 3.2. Identification of Critical Growth Factors Using FFD

**Figure 3:**
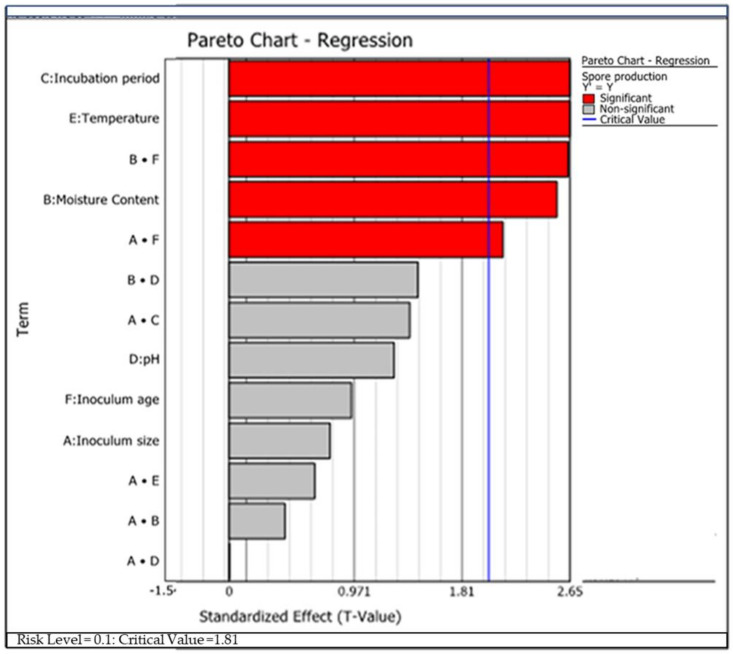
Pareto regression graph indicating the effect of growth factors on conidia production by *T. asperellum* AU131.

**Figure 4:**
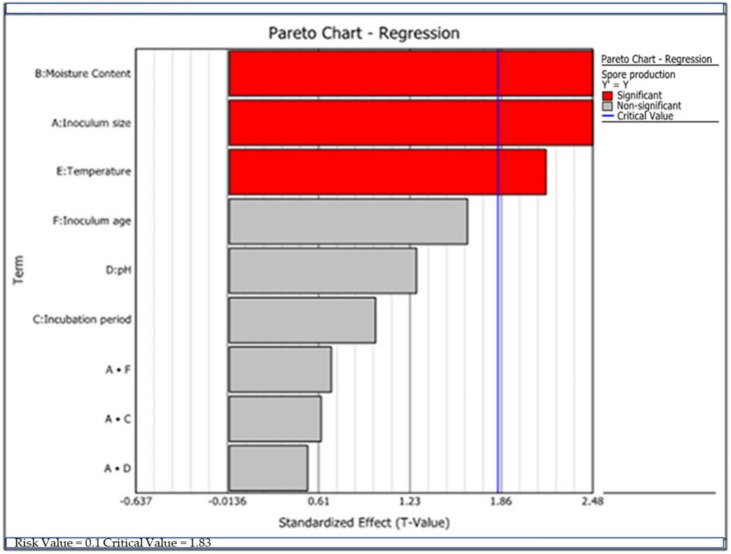
Pareto-regression graph indicating the effect of growth factors on conidia production by *T. longibrachiatum* AU158.

Biomass production of *Trichoderma* species for different growth factors was evaluated statistically based on the results of experimental design (Table 2). The findings of these experiments showed that the significant first-order growth factors that most influenced biomass production of *T. asperellum* AU131 were temperature (E), moisture content (B), and incubation period (C), whereas the most significant second-order growth factors were AF and BF (*p ≤* 0.05) (Table 7). On the other hand, the significant first-order growth factors that most influenced conidia production in *T. longibrachiatum* AU158 were temperature (E), moisture content (B) and inoculum concentration (C) (*p* ≤ 0.05) (Table 8). However, there are no second-order growth factors that influenced biomass production in *T. longibrachiatum* AU158. In both *Trichoderma* species, moisture content and temperature were found to be the most determining factors in conidia production. The order of significance of the tested variables for conidia production by *T. asperellum* AU131 and *T. longibrachiatum* AU158 is presented in Figure 3 and Figure 4, respectively, as a Pareto chart. The significant variables affecting the biomass production of both *Trichoderma* species were considered for further optimization using the statistical model of Box–Behnken RSM.

### 3.3. BBD Approach for Optimization of Conidia Production

According to BBD, batch experiments were carried out with different combinations of the independent variables to determine the combined effects of these factors on biomass/conidia production. The factor levels were defined based on preliminary experiments. A second-order quadratic model was expressed by the following equations to represent conidia production (***Y***) as a function of the incubation period (***C***), moisture content (***A***), and incubation temperature (***B***) for *T. asperellum* AU131; and moisture content (***A***), incubation temperature (***B***) and inoculum concentration (***C***) for *T. longibrachiatum* AU158 (Equations (1) and (2)).

*T. asperellum* AU131 = 8.01 − 0.34*A* − 0.47*B* + 0.23*C* − 0.18*AB* + 0.25*AC* − 0.45*BC* − 0.57*A*^2^ − 0.27*B*^2^ − 0.36*C*^2^ (1)

*T. longibrachiatum* AU158 = 7.06 − 0.25*A* − 0.24*B* + 0.27*C* + 0.34*AB* − 0.29*AC* − 0.11*BC* + 0.26*A*^2^ + 0.20*B*^2^ + 0.17*C*^2^ (2)

In Equations (1) and (2), *A*, *B*, and *C* are independent singular factors, whereas *AB*, AC, and *BC* are interaction factors, and the quadratic terms include *A*^2^, *B*^2^, and *C*^2^.

**Figure 5:**
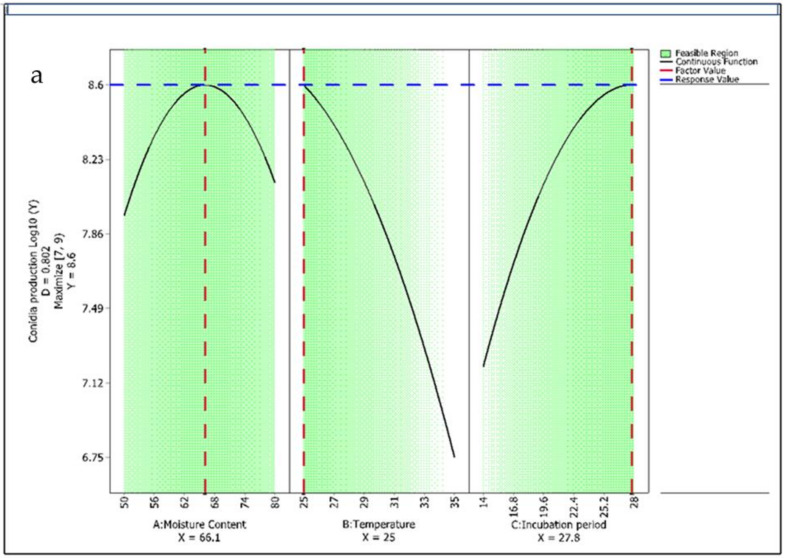

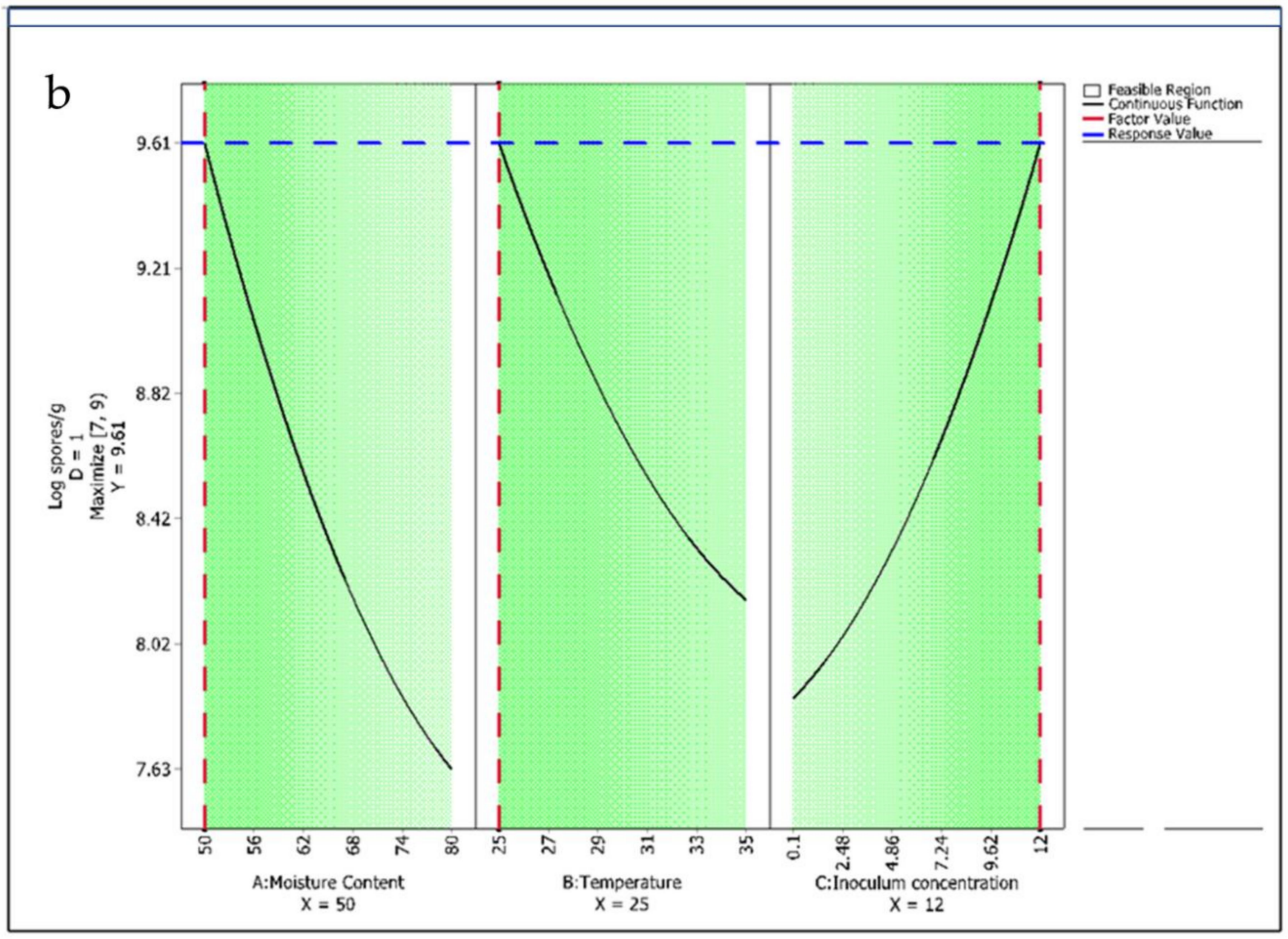
Optimal process conditions for biomass production of (**a**) *T. asperellum* AU131, and (**b**) *T. longibrachiatum* AU158 under SSF.

A BBD matrix of independent variables in coded units and experimental and predicted values of biomass production are shown in Table 5 and Table 6. ANOVA was used to evaluate the statistical significance of the model as well as individual model terms. The influence of three independent factors on the biomass/conidia production was described through the significant coefficient (*p* < 0.05) of the second-order polynomial regression equation. In line with these values, a quadratic model was the most suitable for explaining the experimental data and optimal conditions were achieved with minimum variables (Figure 5). In all cases, a large *F*-value and small *P*-value implied a significant effect of the respective response factors. ANOVA showed that this regression model was highly significant (*p* ≤ 0.05) with *F*-values of 10.38 (*p* = 0.0027) for *T. asperellum* AU131 and 12.01 (*p* < 0.0017) for *T. longibrachiatum* AU158 (Table 9 and Table 10). The multiple correlation coefficients (*R*^2^ values) were 0.9301 (*T. asperellum* AU131) and 0.939 (*T. longibrachiatum*), which indicates good agreement between the experimental and predicted values, showing that 93 and 93.9%, respectively, of the variability in the responses could be well explained by the model. 

**Figure 6:**
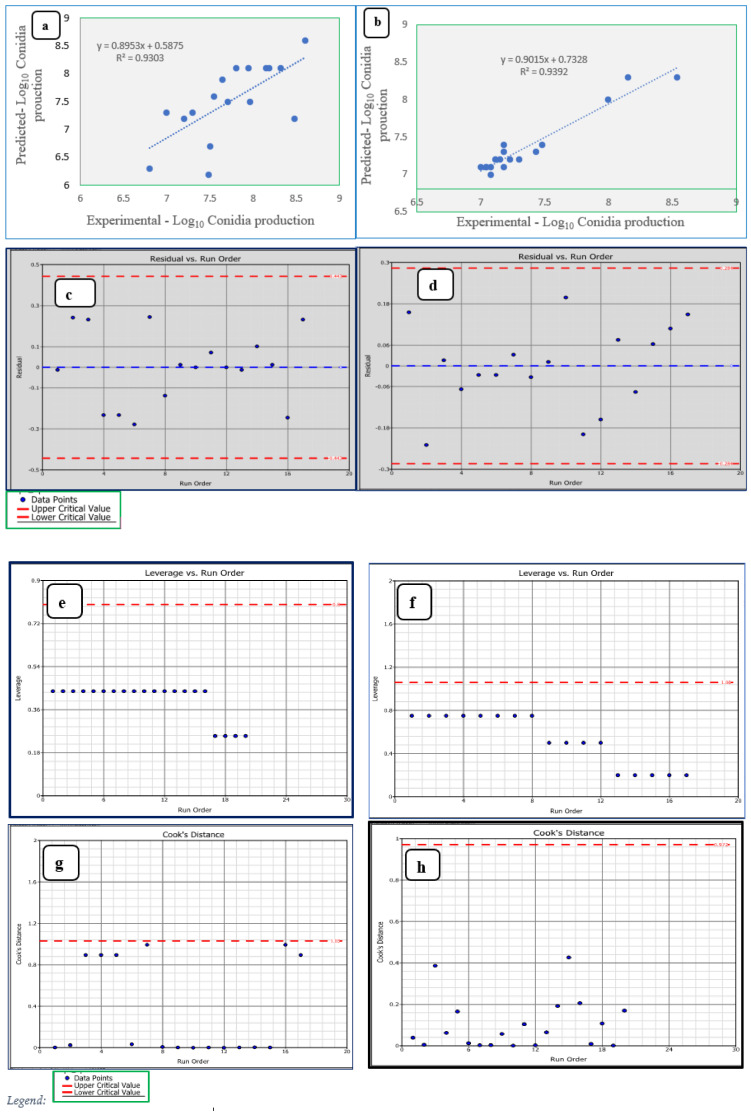
Diagnostic plots for Box–Behnken model adequacy (**a**,**c**,**e**,**g**) for *T. asperellum* AU131 and (**b**,**d**,**f**,**h**) for *T. longibrachiatum* AU158.

The actual *R*^2^ and adjusted *R*^2^ values of both species were close to 1, indicating a high correlation between the experimental and predicted values (Figure 6a,e). The data points were localized close to the diagonal line of fit, indicating a good fit to the model. The lack of fit value was found to be insignificant (*p*-value = 0.186) for *T. asperellum* AU131, suggesting that the model equation was adequate in predicting its biomass production under different combinations of the variables. In contrast, the lack of fit value was found to be significant (*p*-value = 0.0156) for *T. longibrachiatum* AU158, indicating that the model was not well fitted. The maximum experimental and predicted response for biomass production was 8.6 log_10_(conidia/g dry substrate) in *T. asperellum* AU131, indicating strong agreement between them (Table 5). For *T. longibrachiatum* AU158, the maximum experimental response was 8.54 log_10_(conidia/g dry substrate), whereas the predicted response was 8.3 log_10_(conidia/g dry substrate) (Table 6).

It can be concluded from the data in Table 9 and Table 10 that the coefficients of the linear effect of each model term (*A*, *B* and *C*) for both species were significant, suggesting that all three parameters were critical for conidia production. The quadratic effects of the model terms moisture content (*A*^2^), temperature (*B*^2^) and inoculum concentration (*C*^2^) were significant in *T. asperellum* AU131 (Table 9), whereas only moisture content (*A*^2^) and inoculum concentration (*C*^2^) were significant in *T. longibrachiatum* AU158 (Table 10). This implies that the biomass production was affected by these parameters and a small change to their values can affect the process significantly. The coefficients of the cross-product terms *AB* and *AC* were found to be significant in *T. longibrachiatum* AU158, suggesting that there was a strong interaction between moisture content and temperature, as well as between moisture content and inoculum concentration. On the other hand, there was a strong interaction between temperature and time of incubation (BC) in *T. asperellum* AU131.

### 3.4. Process Optimization for Conidia Production

In this study, a high correlation was obtained for the BBD model, indicating that a quadratic polynomial model could be employed to optimize the SSF of both antagonists for maximizing conidia production. To validate the solution suggested by the numerical optimization techniques and to evaluate its accuracy, an experiment was carried out with the suggested optimum values of independent factors. BBD was used to optimize culture conditions using optimal point prediction analysis, which predicted the maximum conidia production of *T. asperellum* AU131 of 8.6 log_10_ (conidia/g dry substrate) at 25 °C and 66.1% moisture content after 27.8 days of incubation (Figure 5a). The optimal point prediction analysis for maximum conidia production of *T. longibrachiatum* AU158 was 9.62 log_10_ (conidia/g dry substrate) at 25 °C and 50% moisture content at 12% inoculum concentration (Figure 5b). The results of this mathematical modeling demonstrated its applicability for predicting the growth rate of *Trichoderma* species under SSF culture conditions. The optimized sets of culture conditions were selected and used for further formulation studies. This approach could be used to predict the impact of controlled environmental conditions on the growth rate of *Trichoderma* species and assist in the formulation of a commercial product.

### 3.5. Accuracy of Box–Behnken Model

The statistical analysis of obtained data revealed that the model was significant (*p* ≤ 0.05). Figure 6a,e shows the experimental values obtained were quite close to the predicted values, representing that the present model successfully enhanced the relationship between the process variables on the response. The residuals and the effect of experimental runs were analyzed by constructing the satisfying fit of the model and it indicated that all the data points place within the limits (Figure 6b,f). The values were less than or equal to 1 for all leverage points, suggesting no unexpected errors in the model (Figure 6c,g). Subsequently, the values of the cook’s distance are in the determined range (Figure 6d,h), and no strong evidence of influential observations in experimental data has been shown. Thus, the RSM model generated in this study satisfied all the necessary arguments for its use in the optimization. Therefore, the application of mathematical modeling was a significant approach to optimize the culture conditions with a smaller number of experiments.

### 3.6. Shelf Life and Viability of Bio-Fungicides

**Figure 7:**
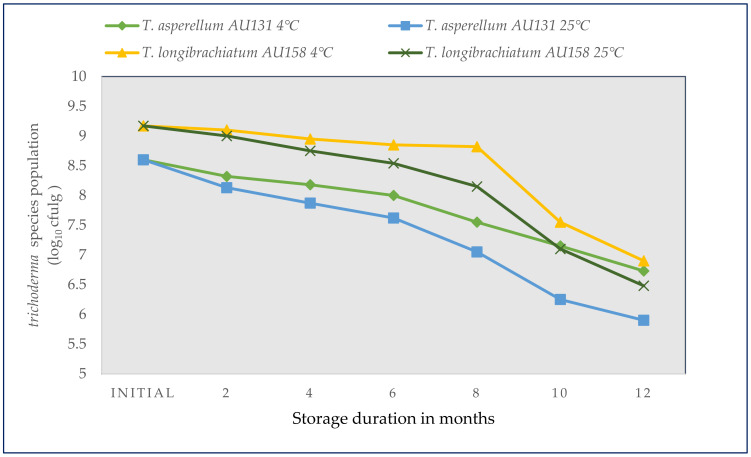
Shelf life and viability of bio-fungicides under two different storage conditions.

The formulated bio-fungicides obtained from the SSF process comprised a homogenous mixture of talc powder and *Trichoderma* spores with 8% moisture content and an initial count of 8.6–9.2 log_10_CFU g^−1^ formulation. The SSF system proved to be applicable for the production of different biocontrol agents. The pattern of population decline in both *T. asperellum* AU131 and *T. longibrachiatum* AU158, in which the maximum initial populations were observed (0.4 × 10^9^ (8.6 log_10_CFU g^−1^) and 1.6 × 10^9^ (9.2 log_10_CFU g^−1^ substrate), respectively), was similar. Although the formulated wettable powder retained the viability of *T. asperellum* AU131 and *T. longibrachiatum* AU158 for a long time, there was a general decline in the number of CFUs with increasing time of storage at both temperatures, with a rapid decrease occurring at room temperature. In other words, both *Trichoderma* species survived better at 4 °C than at 25 °C for 12 months (Figure 7).

The population of the biocontrol fungal species at both temperatures declined slowly until 6 months and then decreased sharply, except for *T. longibrachiatum* AU158 stored at 4 °C, which started to decline at eight months. The population decline continued to 12 months, with a larger decrease in both *T. asperellum* AU131 and *T. longibrachiatum* AU158 at room temperature, and the final populations were counted as 8 × 10^5^ (5.9 log_10_CFU g^−1^) and 3 × 10^6^ (6.48 log_10_CFUg^−1^ substrate), respectively. The decrease in the populations at 4 °C, stored in a refrigerator, started slowly and continued to decline to 12 months, reaching 2.5 × 10^6^ (6.73 log_10_CFU g^−1^) and 8 × 10^6^ (6.9 log_10_CFU g^−1^) for *T. asperellum* AU131 and *T. longibrachiatum* AU158, respectively. Despite the loss of viability of the biocontrol fungi during their incubation in the refrigerator, this population decline was considerably lower compared to the incubation of the same formulated products at room temperature (Figure 7). This study showed that formulations of talc powder and fungal antagonists could represent a practical and effective method for biological control of plant pathogens, e.g., control of CWD caused by *F.* *xylarioides.*

## 4. Discussion

The use of agro-industrial waste for the production of value-added products is a good approach for developing low-cost carriers for the formulation of *Trichoderma*-based bioproducts. It provides avenues for the safe utilization of wastes while reducing the cost and environmental pollution load of waste disposal. Intensive studies are needed to select fermentation substrates that provide large, stable and effective microbial populations for the formulation process [47]. In this study, a broad range of organic materials locally available in Ethiopia was investigated for the growth and multiplication of *T. asperellum* AU131 and *T. longibrachiatum* AU158 in SSF to select suitable agriculture byproducts that favor the production of a high amount of conidia biomass with prolonged viability. Results of the current research showed that *T. asperellum* AU131 and *T. longibrachiatum* AU158 grew on all 14 solid substrates examined and abundantly sporulated on them, but the level of colonization and production of biomass differed among the growth media, most likely reflecting the different ingredients in the organic substrates and the food preference of the *Trichoderma* species. The physicochemical features of organic substrates are known to greatly affect the fermentation process [48]. 

Among the various agro-industrial organic substrates and cereals screened using the OFAT method, wheat bran combined with white rice (2:1 *w*/*w*) was found to support maximum growth of *T. asperellum* AU131 (3.2 × 10^7^ spores/g) and *T. longibrachiatum* AU158 (3.5 × 10^7^ spores/g), significantly (*p ≤* 0.05) higher than for the other substrates. De la Cruz-Quiroz et al. [35] used corn cob as a substrate and a plastic bag as a bioreactor for SSF of a *T. asperellum* strain and showed that the sporulation rate was 1.4 × 10^9^ conidia/g. Surprisingly, rice and wheat bran, which are widely used as organic substrates for the mass production of *Trichoderma* species, enabled high growth of the biocontrol agents examined in our study. One of the most important criteria for selecting a substrate for SSF is the amount of sporulation of the target microorganism. Combination of wheat bran and rice (2: 1 *w/w*) was found to be a suitable raw material for maximum conidia production under SSF, most likely due to the presence of soluble oligosaccharides, nitrogen content, hemicellulose, starches and easily available celluloses, which significantly induce cellulase production [49]. Wheat bran and rice-based substrates are the most common media for the growth and sporulation of many fungi [50]. Both substrates are rich in cellulose, hemicellulose, and lignin and represent a good source of nutrients for the prolific growth and sporulation of *Trichoderma* species [51]. Degradation of cellulose helps *Trichoderma* species to obtain nutrients effectively [52]. Both *T. asperellum* and *T. longibrachiatum* have been found to produce cellulase enzymes [53,54,55], which can effectively degrade the cellulosic content. It is well known that wheat bran is particularly suitable for SSF because of its porosity, allowing good water absorption, which is indispensable for carrying out microbial metabolism. Moreover, in terms of the volume produced, it is a major solid agro-industrial byproduct generated worldwide, including in Ethiopia [56]. Wheat bran promotes fungal growth just as in the natural environmental conditions and requires no additional nutrients for the production of *Trichoderma* spores [18]. Sala et al. [50] used rice husks in SSF of *T. harzianum* and achieved final spore concentrations of up to 2.0 × 10^9^ conidia g^−1^ dry matter. Members belonging to the genus *Trichoderma* are saprophytic fungi, which grow profusely on a wide range of organic substrates in nature [57]. Most commercial products contain *Trichoderma* conidia. Thus, the high productivity of SSF systems is important for the successful production of biocontrol agents [58]. Other reports have also indicated that wheat bran is a suitable substrate for the growth of *T. harzianum*, *T. viride*, *T. koningii, T. asperellum, T. longibrachiatum,* and *T. polysporum* by SSF, supporting the findings of the current study. A lower percentage of lignin may also provide conditions for the easier uptake of cellulose and other inducers required for cellulase production (Table 11). 

In the present study, the maximum conidia production was obtained on WB: WR (2:1 *w*/*w*), WB: SG (2:1 *w*/*w*), sorghum grain, white rice, and sugarcane bagasse, suggesting a feasible approach for using different agro-industrial wastes for the biomass production of these biocontrol agents. The results showed that most of the screened organic substrates could be used to produce a high quantity and quality of *T. asperellum* AU131 and *T. longibrachiatum* AU158 inoculum at low cost. However, other organic substrates screened in this study did not support maximum conidia production of either *Trichoderma* species. Thus, the production of a large number of highly viable conidia was influenced by the type of substrate and presence of a high lignin content (Table 11). Lignin is closely bound to cellulose and hemicellulose, and its functions are to provide rigidity and cohesion to the material cell wall, to confer water impermeability to xylem vessels and to form a physicochemical barrier against microbial attack [59,60]. 

Sachdev et al. [61] reported that sugarcane bagasse + spent tea leaves was the best substrate for the growth of *T. ressei*, whereas for *T. viride* and *T. koningii*, spent tea leaves + wheat bran and for *T. asperellum*, wheat straw + wheat bran were found to be suitable substrates for *T. longibrachiatum*. It is not clear which substrate was best for *T. longibrachiatum.* Analogously, the maximum growth of *T. asperellum* on rice bran was recorded as 10.80 × 10^8^ CFU/g, whereas on sugarcane bagasse only 3.73 × 10^8^ CFU/g was documented after 20 days of incubation [62]. Sargin et al. [63] tested various inexpensive agricultural co-products, including wheat bran, sawdust, rice straw, hazelnut shell, grape marc and cottonseed cake for propagule production of a *T. harzianum* strain and reported that the maximum micro propagule count was achieved with a wheat bran malt sprout mixture. Rayhane et al. [64] studied a fermentation process for enzymes and conidia with a *T. asperellum* strain and scaled up the process from a flask and glass column to a bioreactor. The current study showed that most of the screened organic substrates can be used to produce a high quantity and quality of *T. asperellum* AU131 and *T. longibrachiatum* AU158 inoculum at low cost. In general, these findings suggest that the mass production of *Trichoderma* on different substrates is species-specific according to the different ability to utilize carbon and nitrogen as a source of nutrition.

**Table 11:** Chemical composition of agro-industrial substrates used for SSF.

Substrate	Chemical Composition (% *w*/*w*)					References
	Cellulose	Hemicellulose	Lignin	Ash	Moisture	
Sugarcane bagasse	30.2	56.7	13.4	1.9	4.8	[51]
Wheat straw	32.9	24	8.9	6.7	7	[65]
Coffee husks	23.77	16.7	86	5.4	–	[66]
Potato peel waste	2.2	–	–	7.7	9.9	[67]
Orange peel	9.21	10.5	0.84	3.5	11.9	[68]
Wheat bran	10.9	39	5.08	6.3	12.5	[69]
White rice	0.49	2.85	0.10	3.5	6.4	[70]

Among the six growth factors screened using FFD, incubation temperature, moisture content, inoculum concentration and incubation period were found to be the most significant in affecting the conidia production of both *Trichoderma* species (*p* < 0.05) (Table 8 and Table 9). Individual and combined effects of all the factors in the optimization process were explained by RSM. RSM is beneficial for evaluating multiple parameters and their interactions with a reduced number of experimental trials and aids the improvement, development, and optimization of processes [70]. 

Our results demonstrated its applicability for predicting the growth rate of *T. asperellum* AU131 and T*. longibrachiatum* AU158 under SSF conditions. The results of the statistical analysis indicated that the effects of temperature, initial moisture content of the substrate, inoculum size and incubation time were highly significant (*p* ≤ 0.05). These growth factors were identified as the most influential among physicochemical parameters. Overall, the results suggested that biomass production is markedly affected by these parameters and slight changes in their respective values can affect the process significantly. 

As indicated by the ANOVA results in Table 9 and Table 10, the *F*-values of 10.38 (*T. asperellum* AU131) and 12.01 (*T. longibrachiatum* AU158) indicate that the model was significant at *p* ≤ 0.05, and the probability that the *F*-values were due to noise was only 5%. According to Ferreira et al. [71], comparison between the residual and pure error represents the lack of fit. The *F*-value obtained from the lack of fit was 0.186, indicating that the lack of fit was insignificant (*p* > 0.05) for *T. asperellum* AU131. A non-significant “lack of fit” is acceptable, and therefore the number of experiments was deemed sufficient to evaluate the effects of variables on the response [72]. On the other hand, the regression equation obtained indicated an *R*^2^ value of 0.9301 for *T. asperellum* AU31 and 0.939 for *T. longibrachiatum* AU158. These values demonstrate that the quadratic model was highly significant and could explain about 93% and 93.9%, respectively, of the variability in conidia production by the species. Previous studies have reported ANOVA with high *R*^2^ of 0.9978–0.9070 and *p* < 0.05 [45,73].

The temperature of the SSF culture medium dramatically affected the *Trichoderma* species and conidia production. Both *Trichoderma* species showed an optimal temperature of 25 °C for maximum conidia production (Figure 6a,b). This result is comparable with the results reported by Singh et al. [74], where 25–30 °C was observed as the optimum temperature range for fast growth of *Trichoderma* species. In contrast to the present study, Mohiddin et al. [75] reported that 10 °C was the most favorable temperature for supporting maximum growth of *T. harzianum* compared to 20 °C and 30 °C. The optimal temperature might be the parameter with the highest dependence on the specific strain. Several studies using different substrates have shown an optimal production temperature of 25–28 °C using different *Trichoderma* species [50,76], although conidia production decreased considerably at >30 °C, as also observed in the present study. At higher temperatures, microbial growth is affected and shows less conidia production due to alterations in membrane structure and protein degradation [77]. At lower temperatures, the growth rate of *Trichoderma* species is slow and takes a longer time for conidia production. All these results indicate that temperature is the most important parameter in terms of significance affecting SSF performance, as suggested by Mishra and Khan [31] and Singh et al. [78]. It is known that extremely high or low temperatures denature already synthesized enzymes and other enzymes needed for microbial metabolic activities [79].

The moisture content also determines the growth rate and other physiological activities of *Trichoderma* species. The initial moisture content was found to be significant for conidia production of both *Trichoderma* species analyzed: a lower initial moisture content resulted in higher conidia production. In SSF, microbial growth and product formation occur on the surface of solid particles. The statistical analysis of this study indicated that the optimum moisture content was 50% and 66.1% for *T. asperellum* AU131 and *T. longibrachiatum* AU158, respectively (Figure 6a,b). Santa et al. [80] analyzed the effect of initial moisture and reported an optimal value of 65%, which is similar to that obtained in this study for *T. longibrachiatum* AU158. Even though fungal strains can grow in a wide range of moisture content varying from 40% to 80%, the optimal moisture content may be more dependent on the exact substrate used than on the fungal strain, as suggested by Manpreet et al. [81]. At the same time, the optimum moisture content used in our study was higher than that reported by Mishra et al. [76] (51–54%), which might be due to configuration differences between the reactors used, i.e., packed-bed reactors [76] vs. Erlenmeyer flasks (in this study). Gervais and Molin [82] observed that increased moisture increases aerial mycelial growth of *Trichoderma* species, lowers oxygen transfer and decreases substrate porosity.

A low moisture content in the substrate can reduce nutrient solubility and increase the surface tension of the water layer, hampering fungal growth. If the moisture content is too high, porosity and gas exchange in the substrate are reduced, reducing the efficiency of SSF [18]. This behavior was evidenced in the results presented in Table 5 and Table 6, which show that the lowest initial moisture content (65% and 50%) yielded the highest conidia production (run 9) for *T. asperellum* AU131 and *T. longibrachiatum* AU158 (run 1), respectively. These results agree with Wahid et al. [83] and Aita et al. [84], who investigated optimization of conidia production by SSF with *Trichoderma ressei*. In another study, initial moisture contents from 55 to 65% were evaluated and the best results were obtained with the lowest initial moisture [85]. Therefore, the optimization of initial moisture content in the substrate is essential to maintain the physicochemical characteristics of the substrate and ensure process productivity. 

In the present study, *T. asperellum* AU131 was found to be still in the growth phase after 21 days of incubation, indicating that further increasing the incubation time could result in more conidia production. The maximum conidia production (8.6 log_10_CFU/g dry substrate) was predicted to be after 27.8 days of incubation using optimal point prediction analysis under similar conditions of temperature and moisture. This finding agrees with the results of Sachdev et al. [61], who reported the maximum spore density after 31 days of incubation.

Lastly, an increase in inoculum size from 5% led to a progressive increase in conidia production, reaching the highest value at 10% for *T. longibrachiatum* AU158. The effect of inoculum size on conidia production was also studied by Hamrouni et al. [86], who reported that maximum conidia production was obtained using a 5% inoculum. A smaller inoculum may not be adequate for growth initiation and may delay the lag phase as well as enzyme synthesis [35], whereas a high inoculum size shortens the lag phase but may also increase competition for limiting nutrients due to overcrowded growth of the organism per unit substrate [87]. Therefore, the use of an appropriate inoculum size or dosage is required for healthier fungal propagation and conidia production.

The formulated wettable powder retained the viability of *T. asperellum* AU131 and *T. longibrachiatum* AU158 for a long time. However, there was a general drop in the number of CFUs with the time of storage at 4 and 25 °C, with a rapid decrease occurring at room temperature. High conidia viability is an important aspect of the economics of the production of a *Trichoderma* bio-inoculum, and temperature and moisture are important factors that determine the longevity of a formulation. Although bio-fungicide formulations are dependent on the type of organism being used, the ultimate goal is to ensure that the agent is delivered in a form that is viable, virulent, and has required inoculum potential to be effective in the field [88]. Counting the spores/conidia in the bioproduct does not necessarily reflect the conidia viability as formulations may undergo several stages of processing, including temperature and moisture variation, and storage periods [89]. Thus, CFU/g is a more appropriate measure of conidia viability, as indicated in this study.

In general, despite the similarity of patterns, the viability and population of the antagonist in the talc-based formulation tended to be less affected by storage at 4 °C than at room temperature. The reduction in the shelf life of fungal biocontrol agents during storage is consistent with the findings of other researchers, who reported negative effects of high temperatures and long-term storage on the viability of inocula of *Trichoderma* species [90,91]. Development and preparation of powdery formulations is important for practical application of antagonistic *Trichoderma* species because they can be easily applied as soil and seed treatment for controlling plant diseases under field conditions. Woo et al. [22] reviewed the current applications of *Trichoderma* containing products in agriculture and concluded that 55.3% of *Trichoderma* formulations are commercialized as wettable powders. 

Moreover, the carrier selected should effectively deliver the promising inoculum to the plants. In this study, talc powder was selected because it is chemically defined, less prone to contamination and is internationally accepted [16]. Talc-based formulations of both *Trichoderma* species supplemented with CMC, glycerol and Tween 20 were found to have a stable shelf life of up to six months (Figure 7). Wraight et al. [92] reported that the incorporation of additives in the conidial formulation can have a positive influence on conidial fitness. Addition of glycerol helps to maintain a high moisture content in the formulation and protect the viable propagules from reduced water activity during the shelf life [44]. CMC is an additive that is readily available and has a comparatively steady batch quality since it is a semi-synthetic polymer [93]. In the present study, we concluded that the formulated bioproducts resulted in the retention of viable propagules above 10^6^ conidia/g, which is the minimum requirement for biopesticide application in agriculture. Our previous findings concerning bio-efficacy studies of these bioagents against CWD caused by *F. xylarioides* showed that the bio-fungicides applied under field conditions reduced CWD incidence and severity by 70% and 32% (unpublished data). 

## 5. Conclusions

Wheat bran combined with rice (2:1 *w/w*) was shown to be an efficient agro-industrial waste substrate for mass production of *T. asperellum* AU131 and *T. longibrachiatum* AU158 by SSF. Results obtained by FFD showed that the most significant growth factors influencing the biomass production of both antagonists were temperature, moisture content, inoculum concentration, and incubation period (*p ≤* 0.05). ANOVA statistics from BBD showed that the regression model was highly significant (*p ≤* 0.05) with *F*-values of 10.38 (*p* = 0.0027) and 12.01 (*p* < 0.0017) for *T. asperellum* AU131 and *T. longibrachiatum* AU158, respectively. Under optimal conditions, maximum conidia yield of log_10_ (8.6) and log_10_(9.18) were obtained for *T. asperellum* AU131 and *T. longibrachiatum* AU158, respectively. In the best condition of *Trichoderma* species formulations as a wettable powder, it was possible to maintain conidial viability at room temperature for eight months at concentrations above 10^6^ CFU/g. Mathematical modeling was demonstrated to be a useful approach for predicting the growth rate of both *Trichoderma* species under SSF culture conditions. The proposed approach could be used to predict the impact of controlled environmental conditions on the growth rate of fungal antagonists and assist in the formulation of the commercial product. Thus, industrial-scale production could be achieved with low-cost materials and equipment with a low total capital investment.

## Figures and Tables

**Table 1:** Experimental range, level, and code of independent variables in a two-level FFD.

Independent Variables			Coded Levels			
Factors	Name	Units	−1	0	+1	Std. Dev.
*A*	Inoculum size	%	0.10	5.05	10.00	4.25
*B*	Moisture content	%	50.00	65.00	80.00	12.88
*C*	Incubation period	days	14.00	21.00	28.00	6.01
*D*	pH	–	5.00	6.50	8.00	1.29
*E*	Temperature	°C	25.00	30.00	35.00	4.29
*F*	Inoculum age	days	7.00	10.50	14.00	3.0

**Table 2:** Two-level FFD matrix for screening significant variables for *Trichoderma* species conidia production.

Run	*A*: Inoculum Size (%)	*B*: Moisture Content (%)	*C*: Incubation Period (Days)	*D*: pH	*E*: Temperature (°C)	*F*: Inoculum Age (Days)	Conidia Production log_10_ (*Y*)	
							AUT131	AU158
1	0.1	50	14	5	25	7	7.60	7.52
2	10	50	14	5	35	7	6.48	8.30
3	0.1	80	14	5	35	14	6.18	8.03
4	10	80	14	5	25	14	8.60	7.36
5	0.1	50	21	5	35	14	6.08	8.15
6	10	50	21	5	25	14	8.30	7.08
7	0.1	80	21	5	25	7	6.54	7.40
8	10	80	21	5	35	7	6.48	7.11
9	0.1	50	14	8	25	14	7.18	7.48
10	10	50	14	8	35	14	7.45	8.00
11	0.1	80	14	8	35	7	6.48	7.56
12	10	80	14	8	25	7	6.54	7.72
13	0.1	50	21	8	35	7	6.30	7.66
14	10	50	21	8	25	7	6.64	7.83
15	0.1	80	21	8	25	14	8.70	7.08
16	10	80	21	8	35	14	8.48	7.52
17	5.05	65	17.5	6.5	30	10.5	8.96	7.45
18	5.05	65	17.5	6.5	30	10.5	8.72	7.28
19	5.05	65	17.5	6.5	30	10.5	8.90	7.30
20	5.05	65	17.5	6.5	30	10.5	8.15	7.40

*Y* = Biomass production (conidia/g dry substrate).

**Table 3:** Range, levels and code of independent variables chosen for the BBD of *T. asperellum* AU131.

Variables		Coded Levels			
Factor	Name	−1	0	1	Std. Dev.
*A*	Temperature (°C)	25	30	35	3.54
*B*	Moisture content (%)	35	47.5	60	8.84
*C*	Incubation period (days)	14	21	28	4.95

**Table 4:** Range, levels and code of independent variables chosen for the BBD of *T. longibrachiatum* AU158.

Variables		Coded Levels			
Factor	Name	−1	0	1	Std. Dev.
*A*	Temperature (°C)	25	30	35	3.54
*B*	Moisture content (%)	35	47.5	60	8.84
*C*	Inoculum Concentration (%	0.1	5.05	10	2.80

**Table 5:** BBD matrix together with the experimental and predicted values for optimization of biomass production using *T. asperellum* AU131.

Run	*A*: Moisture Content (%)	*B*: Temperature (°C)	*C*: Incubation Period (Days)	log_10_(*Y*)-Conidia/g Dry Substrate	
				Experimental	Predicted
1	50	25	21	7.64 ± 0.32	7.9
2	80	25	21	7.54 ± 0.5	7.6
3	50	35	21	7.30 ± 0.42	7.3
4	80	35	21	7.48 ± 0.12	6.2
5	50	30	14	7.96 ± 0.15	7.5
6	80	30	14	6.80 ± 0.21	6.3
7	50	30	28	7.70 ± 0.27	7.5
8	80	30	28	7.00 ± 0.33	7.3
9	65	25	14	8.48 ± 0.43	7.2
10	65	35	14	7.20 ± 0.29	7.2
11	65	25	28	8.60 ± 0.14	8.6
12	65	35	28	7.50 ± 0.37	6.7
13	65	30	21	7.80 ± 0.44	8.1
14	65	30	21	8.15 ± 0.25	8.1
15	65	30	21	8.32 ± 0.35	8.1
16	65	30	21	8.18 ± 0.51	8.1
17	65	30	21	7.94 ± 0.40	8.1

Each observed value is represented as mean ± standard deviation.

**Table 6:** BBD matrix along with the experimental and predicted values for optimization of biomass production using *T. longibrachiatum* AU158.

Run	*A*: Moisture Content (%)	*B*: Temperature (°C)	*C*: Inoculum Concentration (%)	log_10_(*Y*)-Conidia/g Dry Substrate	
				Experimental	Predicted
1	50	25	5.05	8.54 ± 0.2	8.3
2	80	25	5.05	7.15 ± 0.33	7.2
3	50	35	5.05	7.23 ± 0.50	7.2
4	80	35	5.05	7.18 ± 0.25	7.4
5	50	30	0.1	7.11 ± 0.45	7.2
6	80	30	0.1	7.43 ± 0.35	7.3
7	50	30	10	8.15 ± 0.60	8.3
8	80	30	10	7.30 ± 0.55	7.2
9	65	25	0.1	7.18 ± 0.12	7.3
10	65	35	0.1	7.08 ± 0.15	7
11	65	25	10	8.00 ± 0.65	8
12	65	35	10	7.48 ± 0.32	7.4
13	65	30	5.05	7.18 ± 0.52	7.1
14	65	30	5.05	7.00 ± 0.18	7.1
15	65	30	5.05	7.08 ± 0.44	7.1
16	65	30	5.05	7.04 ± 0.39	7.1
17	65	30	5.05	7.04 ± 0.24	7.1

Each observed value is represented as mean ± standard deviation.

**Table 7:** ANOVA statistical data of FFD for screening critical growth factors of *T. asperellum* AU131.

Source of Variation	df	Sum of Squares	Mean Squares	F-Test	*p*-Value
Model	8	3.738829	0.467354	5.308835	0.006524 *
*A*: Inoculum size	1	0.052272	0.052272	0.593772	0.045719 *
*B*: Moisture content	1	0.551119	0.551119	6.26036	0.029398 *
*C*: Incubation period	1	1.010127	1.010127	11.474392	0.006063 *
*E*: Temperature	1	0.639942	0.639942	7.269325	0.020799 *
*F*: Inoculum age	1	0.076776	0.076776	0.872126	0.370414
*A • F*	1	0.384261	0.384261	4.364957	0.007212 *
*B • F*	1	0.588263	0.588263	6.68229	0.025363 *
Curvature	1	0.436069	0.436069	4.95346	0.047891 *
Residual	11	0.968365	0.088033		
Lack of fit	8	0.877803	0.109725	3.634814	0.15815
Pure error	3	0.090562	0.030187		
Total	19	4.707194			
* R*^2^ = 90.97%	*R*^2^ (adj) = 65.68%,				

* Significant at *p* ≤ 0.05.

**Table 8:** ANOVA statistical data of fractional factorial design for screening critical growth factors of *T. longibrachiatum* AU158.

Source of Variation	df	Sum of Squares	Mean Squares	F-test	*p*-Value
Model	4	1.772505	0.443126	9.291806	0.000553 *
*A*: Inoculum size	1	0.5776	0.5776	12.111554	0.003357 *
*B*: Moisture content	1	0.6561	0.6561	13.757601	0.002099 *
*E*: Temperature	1	0.216225	0.216225	4.533969	0.050202 *
Curvature	1	0.32258	0.32258	6.764101	0.020067 *
Residual	15	0.71535	0.04769		
Lack of fit	4	0.068075	0.017019	0.289222	0.878921
Pure error	11	0.647275	0.058843		
Total	19	2.487855			
*R*^2^ = 83.24%	*R*^2^ (adj) = 64.61%				

* Significant at *p* ≤ 0.05.

**Table 9:** ANOVA analysis for response surface quadratic model of *T. asperellum* AU131.

Source of Variation	df	Sum of Squares	Mean Squares	F-Value	*p*-Value
Model	9	6.772408	0.75249	10.38162	0.002742 *
*A*: Moisture content	1	0.337862	0.337862	12.27934	0.009073 *
*B*: Temperature	1	2.974082	0.330454	12.01007	0.00163 *
*C*: Incubation period	1	0.451303	0.451303	16.40226	0.048165 *
*A • B*	1	0.045362	0.045362	1.648658	0.222985
*A • C*	1	0.1225	0.1225	2.643568	0.105649
*B • C*	1	0.286375	0.286375	10.40808	0.011758 *
*A • A*	1	0.490038	0.490038	17.81003	0.00349 *
*B • B*	1	0.121269	0.121269	4.407416	0.077298
*C • C*	1	0.563763	0.563763	19.4895	0.02829 *
Residual	7	0.50738	0.072483		
Lack of fit	3	0.3369	0.1123	2.634913	0.186234
Pure error	4	0.17048	0.04262		
Total	16	7.279788			
*R*^2^ = 93.03%		*R*^2^ (adj) = 84.07%			

* Significant at *p* ≤ 0.05.

**Table 10:** ANOVA analysis of the experimental results for the quadratic model of *T. longibrachiatum* AU158.

Source of Variation	df	Sum of Squares	Mean Squares	F-Value	*p*-Value
Model	9	2.974082	0.330454	12.01007	0.001746 *
*A*: Moisture content	1	0.490038	0.490038	17.81003	0.003936 *
*B*: Temperature	1	0.452888	0.452888	16.45984	0.004827 *
*C*: Inoculum concentration	1	0.563763	0.563763	20.4895	0.002711 *
*A • B*	1	0.451303	0.451303	16.40226	0.004871 *
*A • C*	1	0.337862	0.337862	12.27934	0.009937 *
*B • C*	1	0.045362	0.045362	1.648658	0.240004
*A • A*	1	0.286375	0.286375	10.40808	0.014529 *
B • B	1	0.161384	0.161384	5.865382	0.045967 *
C • C	1	0.121269	0.121269	4.407416	0.073938 *
Residual	7	0.192603	0.027515		
Lack of fit	3	0.174755	0.058252	13.05515	0.015595 *
Pure error	4	0.017848	0.004462		
Total	16	3.166685			
*R*^2 =^ 93.92% *R*^2^ (adj) ^=^ 86.10%		*R*^2^ (Pred) = 10.82% Press = 2.82 *S* = 0.166			

* Significant at *p* ≤ 0.05.

## Data Availability

Not applicable.
